# Decreased Expression of PTPN12 Correlates with Tumor Recurrence and Poor Survival of Patients with Hepatocellular Carcinoma

**DOI:** 10.1371/journal.pone.0085592

**Published:** 2014-01-24

**Authors:** Rong-Zhen Luo, Pei-Qiang Cai, Mei Li, Jia Fu, Zhi-Yi Zhang, Jie-Wei Chen, Yun Cao, Jing-Ping Yun, Dan Xie, Mu-Yan Cai

**Affiliations:** 1 Sun Yat-sen University Cancer Center, State Key Laboratory of Oncology in South China, Collaborative Innovation Center for Cancer Medicine, Guangzhou, China; 2 Department of Pathology, Sun Yat-sen University Cancer Center, Guangzhou, China; 3 Medical Imaging and Minimally Invasive Interventional Center, Sun Yat-sen University Cancer Center, Guangzhou, China; Xiangya Hospital of Central South University, China

## Abstract

**Background:**

Protein tyrosine phosphatase non-receptor type 12 (PTPN12), has been identified as a potent tumor suppressor in human cancers and a critical regulator of cell adhesion and migration. However, the PTPN12 expression and its prognostic significance in HCC have not been well elucidated.

**Methodology/Principal Findings:**

In this study, tissue microarray-based immunohistochemistry (IHC) was investigated in an HCC cohort with adjacent liver tissues as controls. The resulting data were analyzed using receiver operating characteristic curves, Spearman's rank correlation, Kaplan-Meier plots and Cox proportional hazards regression modeling. Our results showed that decreased expression of PTPN12 was more frequently observed in HCC tissues compared to the adjacent non-tumorous liver tissues. Further correlation analyses indicated that the decreased PTPN12 expression was closely correlated with tumor recurrence (*P* = 0.015). Univariate analysis showed a significant association between decreased expression of PTPN12 and adverse cancer-specific survival and recurrence-free survival (*P*<0.001). In different subsets of overall patients, PTPN12 expression was also a prognostic indicator in patients with stage I/II or stage III/IV (*P*<0.05). Importantly, multivariate analysis (*P*<0.05) identified PTPN12 expression in HCC as an independent prognostic factor.

**Conclusions/Significance:**

Our findings provide a basis for the concept that PTPN12 protein expression is frequently decreased or lost in human HCC tissues and that decreased PTPN12 expression may represent an acquired recurrence phenotype of HCC and that PTPN12 expression may act as a biomarker of prognosis for patients with HCC.

## Introduction

Hepatocellular carcinoma (HCC) is one of the most lethal malignant cancers worldwide. It has been reported recently that the incidence and mortality of HCC have been increasing [1]. Due to the high prevalence of hepatitis B virus (HBV) infection in Chinese populations, HBV-related liver cirrhosis and/or HCC has become a main disease burden in China [2]. Early detection of HCC allows for curative or palliative treatment with surgical resection or transcatheter arterial chemoembolization [3]. However, because of lack of detectable early symptoms and its insidious onset, most HCC patients were diagnosed at advanced stages, contributing to a relatively low reported 5-year survival rate of approximately 10% [4], [5]. Therefore, the identification of novel genetic biomarkers is of important because this would allow early detection of HCC, provide new therapeutic targets for cancer treatments, and ultimately improve overall survival for HCC patients. Intracellular signaling cascades depend on dynamic phosphorylation events that are tightly controlled by both kinases and phosphatases. Protein tyrosine phosphatases (PTPs) play a crucial role in cellular physiology, signal transduction and carcinogenesis [6], [7]. These PTPs can serve as antagonists to tyrosine kinase signaling, thereby playing an important role in tumor suppression [8], [9]. Protein tyrosine phosphatase non-receptor type 12 (PTPN12), located in 7q11.23, is a member of the PTP family [10]. Previous studies indicated that PTPN12 was a ubiquitously expressed cytosolic PTP and a critical regulator of cell adhesion and migration [11], [12]. Recently, there is an increasing body of evidence that decreased expression of PTPN12 occurs in various human malignancies, including breast cancer, colon cancer, ovarian cancer and esophageal squamous cell carcinoma [9], [10], [13], [14]. However, the expression pattern of PTPN12 and its prognostic significance in HCC have not been well elucidated.

In the current study, we measured the PTPN12 protein expression levels by tissue microarray-based immunohistochemistry (IHC) in an HCC cohort with adjacent liver tissues as controls. Receiver operating characteristic (ROC) curve analysis was conducted to define the cut-off value for separating PTPN12 expression into decreased- and normal-expression groups. The PTPN12 IHC staining results were then correlated with a variety of clinicopathologic parameters and patient follow-up data using various statistical models.

## Materials and Methods

### Ethics statement

The study was approved by the Institute Research Medical Ethics Committee of Sun Yat-sen University. No informed consent (written or verbal) was obtained for use of retrospective tissue samples from the patients within this study, most of whom were deceased, since this was not deemed necessary by the Ethics Committee, who waived the need for consent. All samples were anonymised.

### Patients and tissue specimens

For this study, paraffin-embedded pathological specimens from 248 patients with HCC were obtained from the archives of the Department of Pathology, Sun Yat-sen University Cancer Center, Guangzhou, China, between 1997 and 2008. The cases were selected based on the following criteria: pathological diagnosis of HCC; primary and curative resection for tumor without preoperative or postoperative anticancer treatment; and the availability of resection tissue and follow-up data. The HCC cohort included 220 (88.7%) men and 28 (11.3%) women with a mean age of 47.8 years. The average follow-up time was 31.8 months (median, 26.0 months; range, 1.0 to 86.0 months). Cancer-specific survival (CSS) was defined as the interval between surgery and death of HCC or the last observation taken. For surviving patients, the data were censored at the last follow-up. Deaths from other causes were treated as censored cases. Recurrence-free survival (RFS) was defined as from the date of resection until the detection of tumor recurrence, death or the last follow-up assessment. For RFS analysis, the data were censored for patients without signs of recurrence. The clinicopathologic features summarized in Table 1 include age, sex, hepatitis history, serum alpha-fetoprotein (AFP) level, the presence of cirrhosis, the number of tumors, tumor size, level of tumor differentiation, tumor stage, the extent of vascular invasion and relapse occurrence. Tumor differentiation was determined based on the criteria proposed by Edmonson and Steiner [15]. Tumor stage was defined according to the tumor-node-metastasis (TNM) classification system from the American Joint Committee on Cancer/International Union Against Cancer [16]. The patients were followed every 3 month for the first year and then every 6 months for the next 2 years and finally annually after surgery. The tumor recurrence (including intrahepatic recurrence or metastasis) was detected by ultrasonography, CT or MRI. The time of detection of recurrence was still not known until the patient was dead of HCC, and the time to death was used instead. The Institute Research Medical Ethics Committee of Sun Yat-sen University Cancer Center granted approval for this study.

**Table 1:** Correlation of PTPN12 expression with patients' clinicopathological features in primary hepatocellular carcinomas.

Variable	PTPN12 protein			
	All cases	Low expression	High expression	*P* value*
Age (years)				0.981
≤47.8^†^	123	73 (59.3%)	50 (40.7%)	
>47.8	125	74 (59.2%)	51 (40.8%)	
Sex				0.165
Male	220	127 (57.7%)	101 (40.7%)	
Female	28	20 (71.4%)	8 (28.6%)	
HbsAg				0.709
Positive	216	129 (59.7%)	87 (40.3%)	
Negative	32	18 (56.3%)	14 (43.8%)	
AFP (ng/ml)				0.051
≤20	119	63 (52.9%)	56 (47.1%)	
>20	129	84 (65.1%)	45 (34.9%)	
Liver cirrhosis				0.371
Yes	179	103 (57.5%)	76 (42.5%)	
No	69	44 (63.8%)	25 (36.2%)	
Tumor size (cm)				0.634
≤5	133	77 (57.9%)	56 (42.1%)	
>5	115	70 (60.9%)	45 (39.1%)	
Tumor multiplicity				0.791
Single	140	84 (60.0%)	56 (40.0%)	
Multiple	108	63 (58.3%)	45 (41.7%)	
Differentiation				0.549
I-II	148	90 (60.8%)	58 (39.2%)	
III-IV	100	57 (57.0%)	43 (43.0%)	
Stage				0.319
I-II	173	99 (57.2%)	74 (42.8%)	
III-IV	75	48 (64.0%)	27 (36.0%)	
Vascular invasion				0.185
Absent	170	96 (56.5%)	74 (43.5%)	
Present	78	51 (65.4%)	27 (34.6%)	
Recurrence				0.015
Absent	100	50 (50.0%)	50 (50.0%)	
Present	148	97 (65.5%)	51 (34.5%)	

* Chi-square test; **^†^**Mean age; HBV, hepatitis B virus; HCV, hepatitis B virus; AFP, alpha-fetoprotein.

### Tissue microarray (TMA) construction

Tissue microarrays were constructed in accordance with a previously described method [2]. Briefly, formalin-fixed, paraffin-embedded tissue blocks and the corresponding H&E-stained slides were overlaid for TMA sampling. A senior pathologist (M-Y Cai) reviewed the slides to identify and mark representative tumor areas. Triplicate cylinders (0.6 mm diameter) were punched from the representative tumor areas and from adjacent non-malignant liver tissue from the individual donor's tissue blocks; these cylinders were then re-embedded into a recipient paraffin block at defined positions using a tissue arraying instrument (Beecher Instruments, Silver Spring, MD).

### Immunohistochemistry (IHC)

The TMA block was cut into 4-μm sections and processed for IHC according to the previously described protocol [17]. The TMA slides were de-paraffinized in xylene, rehydrated through a graded alcohol series, immersed in 3% hydrogen peroxide for 10 minutes to block endogenous peroxidase activity, and antigen-retrieval by pressure cooking for 3 minutes in citrate buffer (pH  = 6.0). Then, the slides were preincubated with 10% normal goat serum at room temperature for 30 minutes to reduce nonspecific reactivity. Subsequently, the slides were incubated with rabbit polyclonal anti-PTPN12 antibody (Abcam, Cambridge, MA, 1∶100 dilution) and stored overnight at 4°C. The slides were next washed with PBS (2×5 minutes) and then incubated with a secondary antibody (Envision; Dako, Glostrup, Denmark) for 1 hour at room temperature. Thereafter, the sections were washed with PBS twice for 5 minutes and stained with 3,3-diaminobenzidine (DAB). Finally, the sections were counterstained with Mayer's hematoxylin, dehydrated, and mounted. A negative control was obtained by replacing the primary antibody with normal murine IgG.

### IHC evaluation

Protein expression levels of PTPN12 were evaluated by microscopic examination of stained TMA slides. The presence of cytoplasmic brown granules were considered to be positive for PTPN12 expression. In Brief, the expression pattern was assessed as follows: Each TMA spot was assigned an intensity score from 0–3 (I0, I1–3). Then, the proportion of tumor cells with that intensity was divided by the total number of tumor cells and recorded in 5% increments from 0 to 100 (P0, P1–3). The final H score (range 0–300) was determined by adding the sum of the scores obtained for each intensity and the proportion of the area stained (H score  = I1×P1+I2×P2+I3×P3). PTPN12 expression was assessed by two independent pathologists (R-Z Luo and M Li) who were blinded to the clinicopathological data. All lab methods were used for both tumor and non-tumor specimens.

### Selection of cutoff score

The ROC curve analysis was applied to define a cutoff score for PTPN12 expression by a 0, 1- criterion [18], [19]. Briefly, the sensitivity and specificity for the evaluated outcome were plotted to create various ROC curves. The score localized closest to the point (i.e., 0.0, 1.0) at the maximum sensitivity and specificity was selected as the cutoff score to determine the greatest number of tumors that were correctly classified as having or not having the outcome. To facilitate the ROC curve analysis, the clinicopathologic characteristics were dichotomized as follows: AFP level (≤20 ng/ml vs.>20 ng/ml), tumor size (≤5 cm vs. >5 cm), tumor multiplicity (single vs. multiple), tumor differentiation (I + II vs. III + IV), stage (I + II vs. III + IV), vascular invasion (absence vs. presence), relapse (absence vs. presence) and survival status [death from HCC vs. others (censored, alive or death from other causes)].

### Statistical analysis

Statistical analysis was performed with the SPSS statistical software package (standard version 16.0; SPSS, Chicago, IL). ROC curve analysis was employed to determine the cutoff value for decreased expression of PTPN12. The correlation between PTPN12 expression and the clinicopathologic features of the HCC patients was evaluated by a χ2-test. Univariate and multivariate survival analysis was performed with the Cox proportional hazards regression model. The corresponding Hazard ratio (HR) and 95% CI were taken from Cox regression models. For univariate analysis, survival curves were obtained with the Kaplan-Meier method, and the differences between groups in survival were tested by the log-rank test. Differences were considered significant if the *P*-value from a two-tailed test was <0.05.

## Results

### Selection of the cutoff score for PTPN12 expression

**Figure 1:**
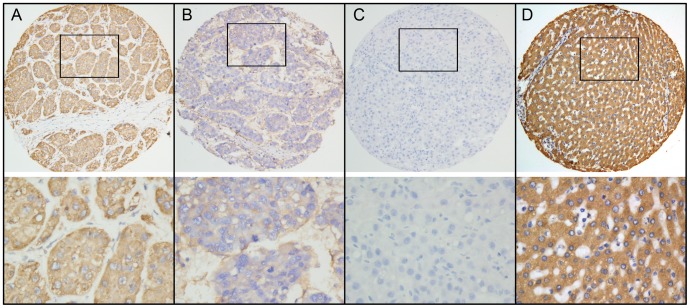
ROC curve analysis was conducted to determine the cutoff score for decreased PTPN12 expression. The sensitivity and specificity for each outcome were plotted: tumor size (**A**), tumor multiplicity (**B**), tumor differentiation (**C**), serum AFP level (**D**), clinical stage (**E**), vascular invasion (**F**), tumor relapse (**G**), survival status (**H**).

**Figure 2:**
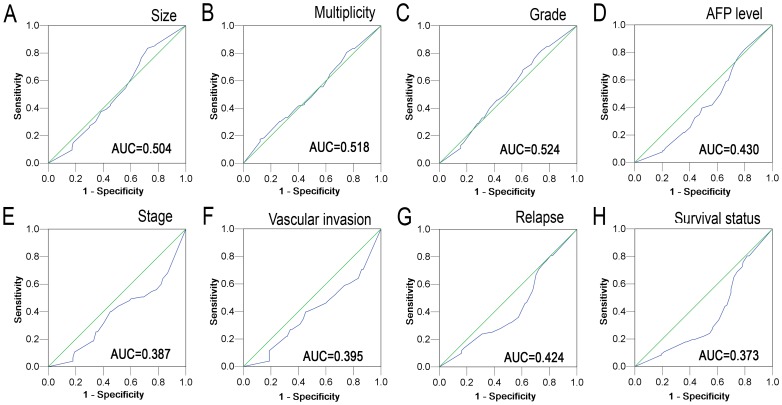
Immunohistochemical staining of PTPN12 protein in HCC and noncancerous liver tissues. **A.** Normal expression of PTPN12 was observed in a HCC case, showing strong diffuse cytoplasmic immunoreactivity of PTPN12 (upper panel, ×100; lower panel, ×400). **B.** A HCC case showed decreased expression of PTPN12, in which all of cancer cells showed weak staining of PTN12 (upper panel, ×100; lower panel, ×400). **C.** Negative expression of PTPN12 was detected in a HCC case (upper panel, ×100; lower panel, ×400). D. Normal expression of PTPN12 was observed in adjacent liver tissues, in which all of cancer cells showed strong staining of PTPN12 (upper panel, ×100; lower panel, ×400).

For PTPN12 IHC staining, immunoreactivity was observed primarily in the cytoplasm within cancerous and noncancerous hepatic cells (Figure 1). The H score of PTPN12 expression ranged from 0 to 300 (Figure 1). To develop an optimal PTPN12 cutoff value for further analysis, we subjected the PTPN12 H score to ROC curve analysis with respect to the clinical features (Figure 2). The ROC curves for each clinicopathologic feature clearly showed the point on the curve closest to (0.0, 1.0), which maximizes both the sensitivity and specificity for the outcome, as described in our previous study [18], [19]. Cancers with scores above the obtained cutoff value were considered to have normal PTPN12 expression, which led to the greatest number of cancers classified, based on the presence or absence of a clinical outcome. As shown in Figure 2, the AUC for survival status had the biggest area. Based on this outcome, we selected a PTPN12 expression score of 140 as the optimal cut-point for survival analysis. According to the ROC curve analysis, decreased expression of PTPN12 could be detected in 147/248 (59.3%) of HCCs and in 102/248 (41.1%) of adjacent liver tissues, respectively. The difference in these H scores was statistically significant (*P*<0.001, Fisher's exact test).

### The relationship between PTPN12 expression and the clinicopathologic features of HCC patients

The correlation between PTPN12 expression and the clinicopathologic features of the HCC patients was determined. The results showed that the decreased expression of PTPN12 was negatively correlated with the tumor recurrence (*P* = 0.005, Table 1). In addition, a strong tendency towards statistical significance (*P* = 0.051, Table 1) has been found between PTPN12 expression and the serum AFP levels.

### The relationship between PTPN12 expression and HCC patient survival

**Figure 3:**
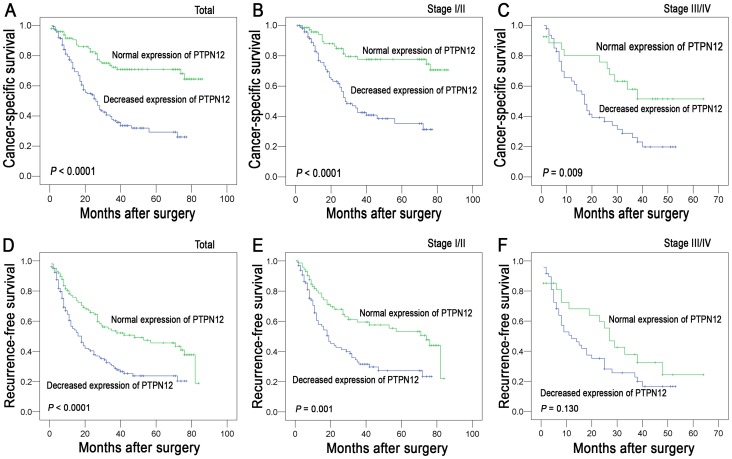
Kaplan-Meier survival analysis of PTPN12 expression in patients with HCC (log-rank test). **A.** The PTPN12 expression and the probability of cancer-specific survival of all patients with HCC: decreased expression, n = 147, normal expression, n = 101. **B.** Probability of cancer-specific survival of the PTPN12 expression in HCC patients at stage I-II: l decreased expression, n = 99, normal expression, n = 74. **C.** Probability of cancer-specific survival of the PTPN12 expression in HCC patients at stage III-IV: decreased expression, n = 48, normal expression, n = 27. **D.** The PTPN12 expression and the probability of recurrence-free survival of all patients with HCC: decreased expression, n = 147, normal expression, n = 101. **E.** Probability of recurrence-free survival of the PTPN12 expression in HCC patients at stage I-II: l decreased expression, n = 99, normal expression, n = 74. **F.** Probability of recurrence-free survival of the PTPN12 expression in HCC patients at stage III-IV: decreased expression, n = 48, normal expression, n  = 27.

The established prognostic factors of the HCC patients' survival were first utilized to test the representativeness of our HCC cases in this study. Univariate analysis demonstrated a significant impact of clinicopathologic prognostic features (i.e., AFP levels, tumor size, tumor multiplicity, clinical stage, vascular invasion and relapse) on the patient survival rates (*P*<0.05, Table 2). Survival assessment revealed that decreased expression of PTPN12 correlated with adverse cancer-specific survival (Cox regression model, hazard ratio: 0.314, 95% CI: 0.202–0.487, *P*<0.001, Table 2). In Kaplan–Meier survival analysis, a highly significant association of decreased PTPN12 expression with shortened patient survival was revealed in HCC patients (Figure 3A, *P*<0.001, Kaplan-Meier method). Stratified survival analysis was also performed with regard to the PTPN12 expression in subsets of the HCC patients at different clinical stages. Our results demonstrated that decreased expression of PTPN12 was a prognostic factor in HCC patients with stage I/II or stage III/IV (*P*<0.05, Figure 3B and 3C, Kaplan-Meier method).

**Table 2:** Univariate analysis of PTPN12 expression and clinicopathologic variables in 248 patients with primary hepatocellular carcinoma (Cox proportional-hazards regression).

Variables	Cancer-specific survival			Recurrence-free survival		
	All cases	Hazard Ratio (95% CI)	*P* value	All cases	Hazard Ratio (95% CI)	*P* value
Age (years)			0.571			0.205
≤47.8*	123	1.0		123	1.0	
>47.8	125	1.004 (0.989–1.020)		125	1.009 (0.995–1.022)	
Sex			0.592			0.532
Female	28	1.0		28	1.0	
Male	220	1.178 (0.647–2.145)		220	0.858 (0.529–1.389)	
HbsAg			0.746			0.326
Negative	32	1.0		32	1.0	
Positive	216	0.911 (0.520–1.596)		216	1.307 (0.766–2.230)	
AFP (ng/ml)			<0.001			<0.001
≤20	119	1.0		119	1.0	
>20	129	3.360 (2.218–5.088)		129	2.520 (1.794–3.541)	
Liver cirrhosis			0.838			0.480
No	69	1.0		69	1.0	
Yes	179	0.958 (0.638–1.439)		179	1.141 (0.791–1.646)	
Tumor size (cm)			<0.001			<0.001
≤5	133	1.0		133	1.0	
>5	115	2.141 (1.463–3.132)		115	1.807 (1.306–2.502)	
Tumor multiplicity			<0.001			<0.001
Single	140	1.0		140	1.0	
Multiple	108	2.369 (1.620–3.465)		108	1.991 (1.435–2.762)	
Differentiation			0. 089			0. 002
I-II	148	1.0		148	1.0	
III-IV	100	1.382 (0.952–2.006)		100	1.661 (1.201–2.298)	
Stage			<0.001			0.002
I-II	173	1.0		173	1.0	
III-IV	75	2.092 (1.428–3.064)		75	1.732 (1.233–2.433)	
Vascular invasion			<0.001			<0.001
Absent	170	1.0		170	1.0	
Present	78	2.696 (1.850–3.928)		78	2.049 (1.464–2.867)	
PTPN12 expression			<0.001			<0.001
Decreased expression	147	1.0		147	1.0	
Normal expression	101	0.314 (0.202–0.487)		101	0.518 (0.366–0.733)	

* Mean age; NR, not reached; HbsAg, hepatitis B surface antigen; AFP, alpha-fetoprotein.

Results in recurrence-free survival analysis were similar to that in cancer-specific survival analysis. Patients with decreased PTPN12 expression also showed a significant trend toward worse recurrence-free survival compared to that with normal PTPN12 expression (hazard ratio: 0.518, 95% CI: 0.366–0.733, *P*<0.001, Table 2, Cox regression model; Figure 3D, Kaplan-Meier method). Additionally, stage-match survival analysis showed that PTPN12 expression was a predictor for recurrence-free survival in stage I/II (*P = *0.001, Figure 3E), and a tendency towards statistical significance was found in stage III/IV (*P = *0.130, Figure 3F, Kaplan-Meier method).

### Independent prognostic factors of HCC

We next analyzed clinical features (including AFP level, tumor size, tumor multiplicity, stage and vascular invasion) and PTPN12 expression that displayed significant impact on patient survival based on univariate analyses with Cox proportional hazards model (Table 3). We found that decreased PTPN12 expression was a prognostic factor for poor cancer-specific survival and recurrence-free survival of HCC patients (*P*<0.001, Table 3) independent of serum AFP levels, tumor size, multiplicity, clinical stage and vascular invasion as evidenced by multivariate analysis. Of other features, AFP level, tumor size, tumor multiplicity and vascular invasion were found to be independent prognostic predictors for poor cancer-specific survival and recurrence-free survival (*P*<0.05, Table 3).

**Table 3:** Cox multivariate analyses of prognostic factors on patients' survival.

Variables	Hazard ratio	95% CI	*P* value
**Cancer-specific survival** *			
AFP, ng/ml (≤20*v* >20)	2.498	1.619– 3.855	<0.001
Tumor size, cm (≤5*v* >5)	1.516	1.021– 2.249	0.039
Tumor multiplicity (single *v* multiple)	1.760	1.182– 2.621	0.005
Stage (I*-*II *v* III*-*IV)	1.217	0.752– 1.969	0.424
Vascular invasion (absent *v* present)	1.929	1.197– 3.107	0.007
PTPN12 expression (decreased *v* normal)	0.353	0.226– 0.552	<0.001
**Recurrence-free survival^†^**			
AFP, ng/ml (≤20*v* >20)	2.481	1.601– 3.845	<0.001
Tumor size, cm (≤5*v* >5)	1.507	1.012– 2.243	0.043
Tumor multiplicity (single *v* multiple)	1.761	1.182– 2.623	0.005
Tumor differentiation (I*-*II *v* III*-*IV)	1.045	0.711– 1.536	0.823
Stage (I*-*II *v* III*-*IV)	1.218	0.752– 1.972	0.422
Vascular invasion (absent *v* present)	1.916	1.184– 3.100	0.008
PTPN12 expression (decreased *v* normal)	0.352	0.225– 0.551	<0.001

* The total number of patients and total number of events in this model were 248 and 112, respectively; **^†^**The total number of patients and total number of events in this model were 248 and 148, respectively; CI, confidence interval; AFP, alpha-fetoprotein.

## Discussion

Despite improvements in surveillance and treatment strategies, the prognosis of HCC remains unsatisfactory because of the high incidences of recurrence and distant metastasis [20], [21]. At present, the current pTNM stage and histological grading systems are established and useful prognostic indicators for HCC. However, patients with the same pTNM stage and/or histological grade of HCC often demonstrate considerable variability in tumor recurrence and metastasis. The tumor heterogeneity and the accumulation of genetic and epigenetic alterations probably lead to the prognostic variability of individuals with HCC [22]. Thus, there is an urgent need for new objective strategies that can effectively distinguish between patients with favorable or unfavorable prognoses in the same stage and/or grade. Although previous studies unraveled many aberrantly expressed genes in HCC [23]–[25], the novel molecular markers that can identify tumor recurrence and aid risk assessment remain substantially limited.

Protein tyrosine phosphorylation plays a central role in cellular physiology and in cancer [6]. Also, PTPs regulate the equilibrium of tyrosine phosphorylation and can serve as antagonists to TK signaling to play a important role in tumor suppression [26]. PTPN12, one member of the PTP family, has been identified as a potent tumor suppressor in human breast cancer and loss of PTPN12 phosphatase activity leads to aberrant acinar morphogenesis and cellular transformation in mammary epithelial cells [9]. In recent years, decreased expression of PTPN12 has been found in several human cancers and correlated with the aggressiveness and/or poor prognosis of breast cancer and esophageal cancer [9], [10], [13], [14]. However, expression of the PTPN12 protein in HCC and its clinicopathologic/prognostic significance in HCC are still unclear. Therefore, we employed TMA-based IHC to examine the expression status of PTPN12 in HCC and determine its potential impact on HCC tumorigenesis and prognosis.

In the current study, we first found that decreased PTPN12 expression was more frequently observed in HCC tissues than in the adjacent liver tissues and that the differences in PTPN12 protein expression from HCC tissues to adjacent liver tissues were statistically significant. Similar results were also found in breast cancer and esophageal squamous cell carcinoma [14], [27], [28]. Yuan Xunyi et al reported that expression of PTPN12 protein was down-regulated in 32.0% of breast cancer tissues and 11.3% of noncancerous breast tissues [28]. Additionally, western blotting result indicated that the expression of PTPN12 protein was lower in esophageal cancer tissues than that in adjacent esophageal tissues [14]. In our study, we demonstrated that decreased PTPN12 expression was closely correlated with tumor recurrence, suggesting that PTPN12 could suppress the formation and proliferation of HCC. Additionally, analysis of the association of PTPN12 expression with clinicopathologic characteristics indicated that low expression of PTPN12 was related to lymph node metastasis, which was consistent with the evidence provided by Tingting Sun et al [9], [27]. Our current findings support that the critical role of PTPN12 as a tumor suppressor in the development and progression of HCC. Collectively, these data suggest that PTPN12 functions as a suppressor of malignant transformation and may be inactivated in cancers.

The most important finding of the present study is the prognostic significance of the PTPN12 expression in HCC. We found that the decreased expression of PTPN12 was a strong and independent predictor of shortened cancer-specific and recurrence-free survival, as evidenced by univariate and multivariate analyses. Importantly, a stratified survival analysis of HCC according to the clinical stage showed that PTPN12 expression was closely correlated with HCC patient survival. Our results suggest that the decreased PTPN12 expression in HCC may facilitate an increased malignant feature and/or a worse survival in this tumor type. With regard to the prognostic impact of PTPN12 on other human cancers, similar findings have been reported in breast cancer and esophageal cancer [14], [27].

As we know, the loss of cell-cell adhesion increases invasion and metastasis, which is an important step in cancer progression. The PTPN protein is thought to act as an important regulator in controlling cell adhesion, motility, and metastasis by interacting with and inhibiting multiple oncogenic tyrosine kinases [29]. Thus, we postulated that PTPN12 may be maintained at normal expression levels in normal liver cells, which may balance cell behaviors, including proliferation, differentiation, mitotic cycle and apoptosis. However, during the course of HCC tumorigenesis, PTPN12 might be compromised in HCC by deletion, inactivating sequence variants or loss of expression. The mechanisms on the potential role of PTPN12 in cancer have been explained in recent investigations. Previous study showed that PTPN12 was a negative regulator of ovarian SKOV-3 cell motility through the control of FAK phosphorylation and its down-modulation by HER2 [10]. Recently, it has been suggested that PTPN12 functions as a suppressor of epithelial cell motility by controlling Rho GTPase activity and the assembly of adherent junctions in colon cancer cells [13]. It has been well established that promoter hypermethylation is one important molecular mechanism leading to gene silencing of tumor suppressor genes. Yuan Xunyi et al. reported that promoter CpG island hypermethylation was found to occur much more frequently in cell lines or specimens with low PTPN12 expression, indicating that it is potentially an important mechanism underlying PTPN12 down-regulation [28]. Although the correlation of PTPN12 expression in cancer and patient survival has been the focus of various studies, the underlying mechanism by which PTPN12 affects prognosis remains elusive and will require future investigation.

In summary, our findings provide a basis for the concept that PTPN12 protein expression is frequently decreased or lost in human HCC tissues and that decreased PTPN12 expression may represent an acquired recurrence phenotype of HCC. More importantly, our study introduces decreased PTPN12 expression as a new adverse independent prognostic factor in HCC. Our findings are potentially significant because it may help us to target a subset of HCC patient population for more aggressive postsurgical anticancer therapies.
